# Molecular generation strategy and optimization based on A2C reinforcement learning in de novo drug design

**DOI:** 10.1093/bioinformatics/btad693

**Published:** 2023-11-16

**Authors:** Qian Wang, Zhiqiang Wei, Xiaotong Hu, Zhuoya Wang, Yujie Dong, Hao Liu

**Affiliations:** College of Computer Science and Technology, Ocean University of China, Qingdao, Shandong 266100, China; College of Computer Science and Technology, Ocean University of China, Qingdao, Shandong 266100, China; College of Computer Science and Technology, Ocean University of China, Qingdao, Shandong 266100, China; Center for High Performance Computing and System Simulation, National Laboratory for Marine Science and Technology, Qingdao, Shandong 266237, China; Marine Big Data Center of Institute for Advanced Ocean Study, Ocean University of China, Qingdao, Shandong 266100, China; College of Computer Science and Technology, Ocean University of China, Qingdao, Shandong 266100, China

## Abstract

**Motivation:**

In the field of pharmacochemistry, it is a time-consuming and expensive process for the new drug development. The existing drug design methods face a significant challenge in terms of generation efficiency and quality.

**Results:**

In this paper, we proposed a novel molecular generation strategy and optimization based on A2C reinforcement learning. In molecular generation strategy, we adopted transformer-DNN to retain the scaffolds advantages, while accounting for the generated molecules’ similarity and internal diversity by dynamic parameter adjustment, further improving the overall quality of molecule generation. In molecular optimization, we introduced heterogeneous parallel supercomputing for large-scale molecular docking based on message passing interface communication technology to rapidly obtain bioactive information, thereby enhancing the efficiency of drug design. Experiments show that our model can generate high-quality molecules with multi-objective properties at a high generation efficiency, with effectiveness and novelty close to 100%. Moreover, we used our method to assist shandong university school of pharmacy to find several candidate drugs molecules of anti-PEDV.

**Availability and implementation:**

The datasets involved in this method and the source code are freely available to academic users at https://github.com/wq-sunshine/MomdTDSRL.git.

## 1 Introduction

It is important to design effective and high-quality compounds, but the process is time-consuming and expensive for the new drug development. Molecular design (Valverde 2001) is usually to generate compounds with specific chemical properties and pharmaceutical potential based on ligand or target structure. Effective molecular design approaches facilitate the rapid discovery of compounds with desired properties, such as high bioactivity, low toxicity and excellent synthetic properties. Such methods are widely employed in the new drug development, including lead compound discovery and molecular structure optimization. However, the existing drug molecular design methods face a significant challenge in terms of generation efficiency and quality. There is still a huge gap between chemically generated molecules and the actual requirements for drug development. The fundamental difficulty stems from the need to systematically establish a correlation mechanism between molecular structure and a variety of drug properties.

Molecular structure determines the pharmacological properties and functions of compounds, therefore, molecular characterization is a fundamental problem in drug computing. In terms of intuitiveness, the 3D structural representation of drug molecules can provide spatial configuration information, facilitating the understanding of the molecule’s spatial properties and interactions (Ragoza *et al.* 2020, Luo et al. 2021). But the accuracy of its prediction results can be affected by factors such as molecular conformational changes and computational resources. Another representation is the molecular graph, which represents the molecule as a graph of nodes and edges, used to analyze the chemical structure and properties of drug molecules (Schütt *et al.* 2017, Li *et al.* 2018, Mercado *et al.* 2021). But cannot express the molecule’s stereochemistry and spatial properties, which can affect applications such as virtual screening and activity prediction. However, the most commonly used representation is the SMILES representation, which has the advantages of simplicity, easy storage, and transmission, but may suffer from ambiguity and partial loss of chemical information (Blaschke *et al.* 2020). And current existing methods can use the molecule’s atomic and bond information to avoid problems in SMILES representation (Scarselli *et al.* 2008).

Regarding the design methods, deep learning techniques have found extensive application in molecular design. A series of methods based on recurrent neural network (RNN), variational autoencoder (VAE), generation adversary network (GAN) and reinforcement learning (RL) have realized the generation of candidate drugs for specific targets. RNN was a widely used neural network structure, which showed a strong processing ability in the drug molecular design (Merk *et al.* 2018, Moret *et al.* 2020). The RNN-based generation model uses low-dimensional vector representation of molecular character sequences to calculate hidden states at each time step to output probability distributions (Olivecrona *et al.* 2017, Popova *et al.* 2018). But, this method only considers random combination of fragments or de novo design, ignoring the advantages of molecular scaffolds. The concept of variational automatic encoder was first proposed at the end of 2013 (Kingma and Welling 2019). The VAE-based molecular generation models map the input molecules to a Gaussian distribution in the latent space using an encoder and decoder, aiming to generate a latent variable distribution similar to the original molecular data by minimizing the reconstruction loss and KL divergence loss (Bjerrum and Sattarov 2018, Dai *et al.* 2018, Simonovsky and Komodakis 2018). GAN could be combined with multi-task learning to optimize different properties of compounds for joint training, so as to complete the multi-objective molecular generation task (Dong *et al.* 2020, Li *et al.* 2021). The GAN-based molecular generation model optimizes the generator and discriminator loss functions by mutual competition, achieving a mapping from random noise space to molecular space to generate new molecules with desired properties (Guimaraes *et al.* 2017, Yu *et al.* 2017, De Cao and Kipf 2018). Reinforcement learning could flexibly adjust different parameters to adapt to multiple tasks in drug computing (Cherti *et al.* 2017, Popova *et al.* 2018, Wang *et al.* 2021). The RL-based molecular generation model follows the Markov decision process, interacting with the chemical space, taking actions to generate new molecules, and evaluating performance by reward function. By constantly updating the policy function, new molecules with superior properties can be generated. Whether it is based on VAE, GAN, or RL methods, the evaluation mechanism for molecule generation is limited to the enumeration of simple properties which cannot be compatible to consider the key druggable properties such as physicochemical properties and bioactivity. Moreover, the scale of molecular docking calculation used for evaluating molecular bioactivity is also extremely large.

Aiming at the above question, we propose a novel molecular generation strategy and optimization based on A2C reinforcement learning. The main contributions of our approach are the following:

1. The generation strategy adopts transformer-DNN to retain the scaffolds advantages, while accounting for the generated molecules’ similarity and internal diversity by dynamic parameter adjustment, further improving the overall quality of molecule generation.
2. In molecular optimization, heterogeneous parallel supercomputing is introduced for large-scale molecular docking based on message passing interface (MPI) communication technology to quickly obtain bioactivity information, thereby improving the efficiency of drug design.
3. Experiments show that our model can generate high-quality molecules with multi-objective properties at a high generation efficiency, with effectiveness and novelty close to 100%.

## 2 Materials and methods

**Figure 1.**
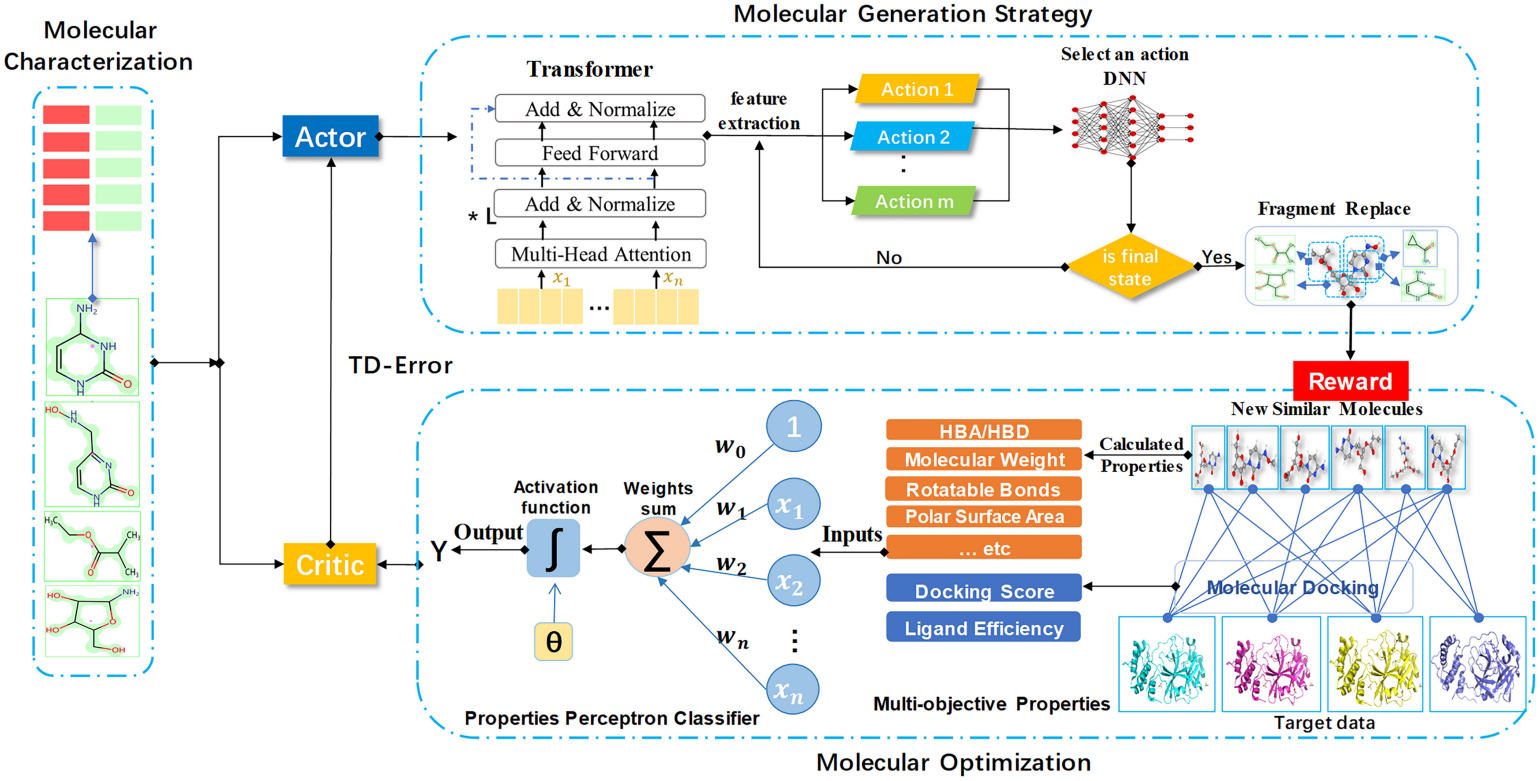
Molecular design framework based on A2C reinforcement learning.

This paper presented a novel molecular generation strategy and optimization by combining transformer-DNN, heterogeneous parallel supercomputing and reinforcement learning. As shown in Fig. 1, we integrated molecular characterization, generation strategy and molecular optimization into the framework based on actor-critic reinforcement learning. The specific submodules will be described in detail below.

### 2.1 Molecular characterization based on the balanced binary tree

Molecular characterization is a fundamental problem in drug design. It directly affects the construction of a clear correlation between molecular structure and drug properties. Therefore, we proposed the molecular characterization based on balanced binary tree.

#### 2.1.1 Fragment combination library construction

We constructed a virtual fragment combinatorial library by fragmenting compounds. The molecules were partitioned into ring structures, side chains and linkers. To achieve this, single bonds extending from a ring atom were cleaved, and the corresponding attachment points were recorded for subsequent assembly steps.

#### 2.1.2 Fragment encoding based on balanced binary tree

For the fragment combination library constructed previously, we calculated the similarity between fragments and then encoded fragments to achieve molecular fragment data preprocessing.

1. Fragment similarity calculations
  In the method, through encoding all fragments as binary strings, similar fragments got similar encodings. Specifically, we employed the Tanimoto-MCS (TMCS)similarity for measuring the similarity, its formula was defined as follows:
  (1) $$\text{TMCS}\left(M_{1},M_{2}\right)=\frac{\text{mcs}\left(M_{1},M_{2}\right)}{a\left(M_{1}\right)+a\left(M_{2}\right)-\text{mcs}\left(M_{1},M_{2}\right)}$$
  Here, $\mathrm{TMCS}(M_{1},M_{2})$ was the number of atoms in the maximum common substructure of molecules $M_{1}$ and $M_{2}$, $a(M_{1})$ and $a(M_{2})$ were the number of atoms, respectively.
2. Fragments encoding
  When constructing the tree, we calculated similarities among all fragments using Equation (1). Subsequently, pairs of fragments were created in a stepwise, bottom-up fashion, starting with the two most similar fragments. This procedure was iterated, and the two pairs containing the most similar fragments were amalgamated into a new tree with four branches. The computed similarity between two subtrees was determined as the highest similarity between any two fragments within those subtrees. This joining process was reiterated until all fragments were consolidated into a unified tree.

All fragments were encoded into binary strings by constructing a balanced binary tree that was based on fragments similarity calculations. Afterwards, each fragment used this tree to generate binary strings. The fragments encodings were determined by the paths from the root to the leaves. Every branching to the left will add a one (“1”) to the end of the encoding and every branching to the right will add a zero (“0”).

### 2.2 Molecular generation strategy based on transformer-DNN

Molecular characterization is the foundation, so the molecular generation strategy is the core of drug design. Molecular generation is able to generate new compounds by performing fragment replacement on an initial set of lead compounds.

**Figure 2.**
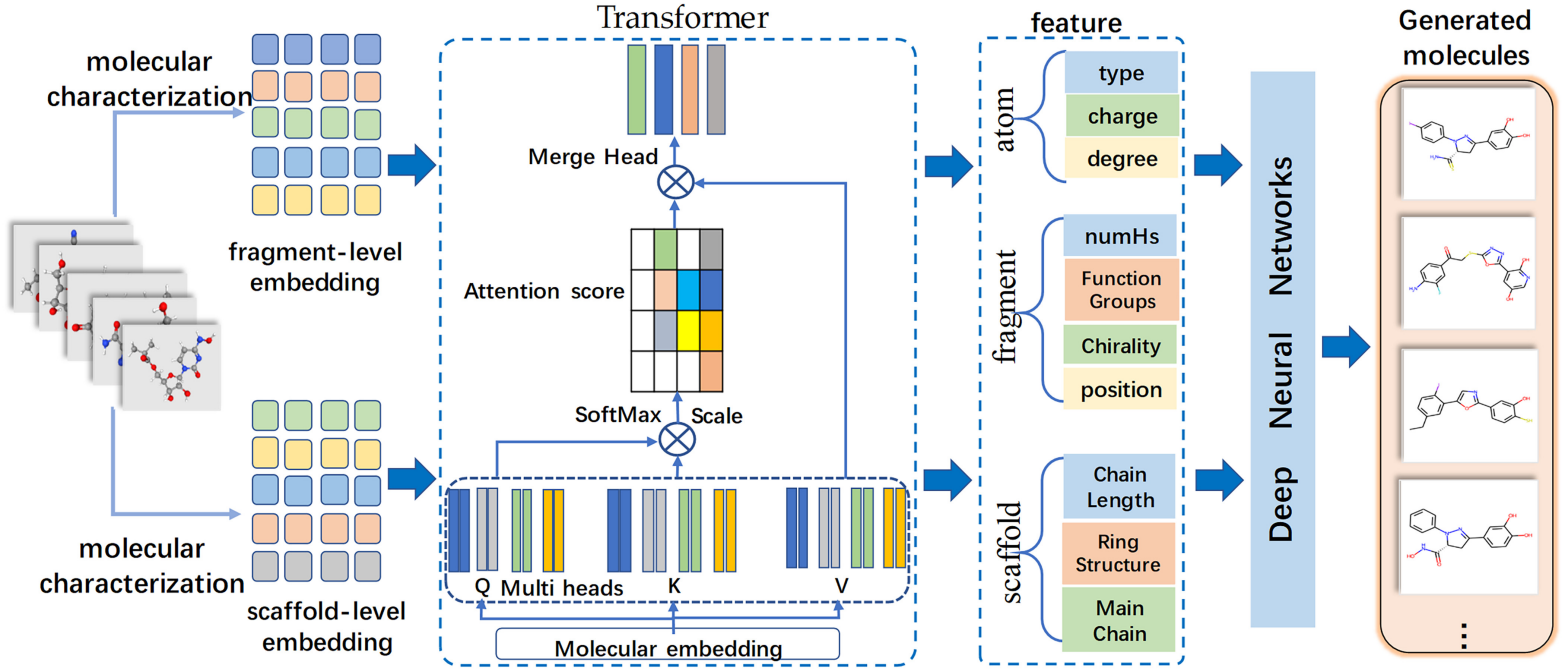
Molecular generation process based on transformer-DNN.

In our method, the generation strategy adopted the transformer-DNN to identify one or more fragments for replacement. Firstly, the molecular sequence was preprocessed through molecular characterization to obtain fragment level embedding with the position information and scaffolds level embedding, which served as the input to the transformer-DNN. Then, using the transformer, the fragments importance was obtained by calculating the attention coefficient of each fragment. This process preserved the advantages of the molecular scaffolds and output a vectorized feature representation of a molecule with different fragment associations. Finally, the concatenated output was classified by the DNN, which estimated the probability distribution of which fragments to replace and with what fragments, as illustrated in Fig. 2.

The molecule’s multi-granularity information features were processed through a transformer layer. This transformer encoder comprised multi-head attention layers, position feedforward network (FFN), residual connection layers and normalization layers. The multi-head attention layer consisted of multiple attention heads, with each head performing linear transformations before calculating attention weights. Different trainable parameter sets were used for each attention head to capture distinct relationships between fragment sequences. This allowed learning of internal molecular structures through attention-weighted calculations. Specifically, the input of the multi-head attention layer consisted of three vector sequences: $W^{Qh},W^{Kh},W^{Vh}\in R^{d_{f}\times d_{p}}$, where $l_{1}$ and $l_{2}$ were the lengths of the first and second fragment sequences, respectively. Next, for the *h*th head, three weight matrices $W^{Qh},W^{Kh},W^{Vh}\in R^{d_{f}\times d_{p}}$ were used to map these three inputs to a lower dimension $d_{p}$. The attention function was then performed:


(2) $$A^{h}=\text{Attention}\left(Q^{h},K^{h},V^{h}\right)$$

where $Q^{h}=QW^{Qh},K^{h}=KW^{Kh},V^{h}=VW^{Vh}$, the attention function produced an array for each vector in the query sequence Q. $A^{h}\in R^{l_{1}\times d_{f}}$ represented the output of the attention function. Then, the output of the multi-head attention layer was obtained through a concatenation of linear transformations from attention heads:


(3) $$\text{MultiHead}(Q,K,V)=\text{Con}\left(A^{1},A^{2},\ldots ,A^{H}\right)W^{O}$$

Here, *H* represented the number of heads, and $W^{O}\in W^{Hd_{p}\times d_{f}}$ was the trained weight matrix. Subsequently, the output of the multi-head attention layer was fed into a feedforward neural network. The feedforward neural network consisted of two linear layers, with the first layer utilizing ReLU as an activation function. The position FFN transformed each vector in the sequence in a uniform manner, as illustrated below:


(4) $$\mathrm{PFN}\cdot (X)=\max\left(0,XW_{1}+b_{1}\right)W_{2}+b_{2}$$

Then, the multi-head attention and the feedforward neural network were connected through residual connections and normalization. After the encoding transformation, we can obtain features for each molecular sequence:


(5) $${\hat{F}}_{f}^{T}=\text{Transformer}\left(F_{f}^{T}\right)$$

(6) $${\hat{F}}_{s}^{T}=\text{Transformer}\left(F_{s}^{T}\right)$$

where ${\hat{F}}_{f}^{T}$ represented fragment-level features, and ${\hat{F}}_{s}^{T}$ represents scaffold-level features of the molecular sequence.

### 2.3 Multi-objective optimization of molecular properties

Molecular generation strategy is the core, in that way molecular optimization is the key in the drug design. In this article, we added the evaluation of physical and chemical properties and molecular docking to the reward of reinforcement learning, the latter of which was used to add biological activity constraints. Considering the computational scale of molecular docking, we had adopted parallel supercomputing, greatly accelerating the speed of molecular generation.

Preliminary generated molecules were screened for toxicity by checking for the presence of known toxic groups. In addition, molecular weight (MW), effectiveness (E), lipophilicity (LogP) and polar surface area (PSA) also affected the pharmacokinetic properties. Only those molecules satisfying the specified range of properties had excellent pharmacokinetic properties. The calculation formula was as follows:


(7) $$P_{k}^{l}\leq p_{k}(s)\leq P_{k}^{u},k\in E,MW,\;\text{log}\;P,\mathrm{PSA},T,LE$$

where *k* represented multiple properties, *l* and *u* were the set range of molecular properties.

We used large-scale molecular docking to obtain docking scores, and then input them into a perceptron classifier for bioactivity prediction. Docking score in each epoch needed to perform docking calculations between the generated compounds in batches and specific target structures, which was obviously a time-consuming operation. To address this issue, we introduced MPI parallel computing based on the heterogeneous parallel supercomputing. In this process, the master process was responsible for reading protein target file data and analyzing target sequences. It intelligently distributed the data to computing nodes within the sub-communication domain based on the relationship between the number of protein targets and the number of worker processes. The worker processes handled the reading of ligand small molecule file data and broadcast it to all computing nodes, while also monitoring the overall program’s execution. The computing nodes were tasked with the actual molecular docking process, and once suitable docking conformations were found, they wrote the docking results to a file and sent a signal to the master process.

**Figure 3.**
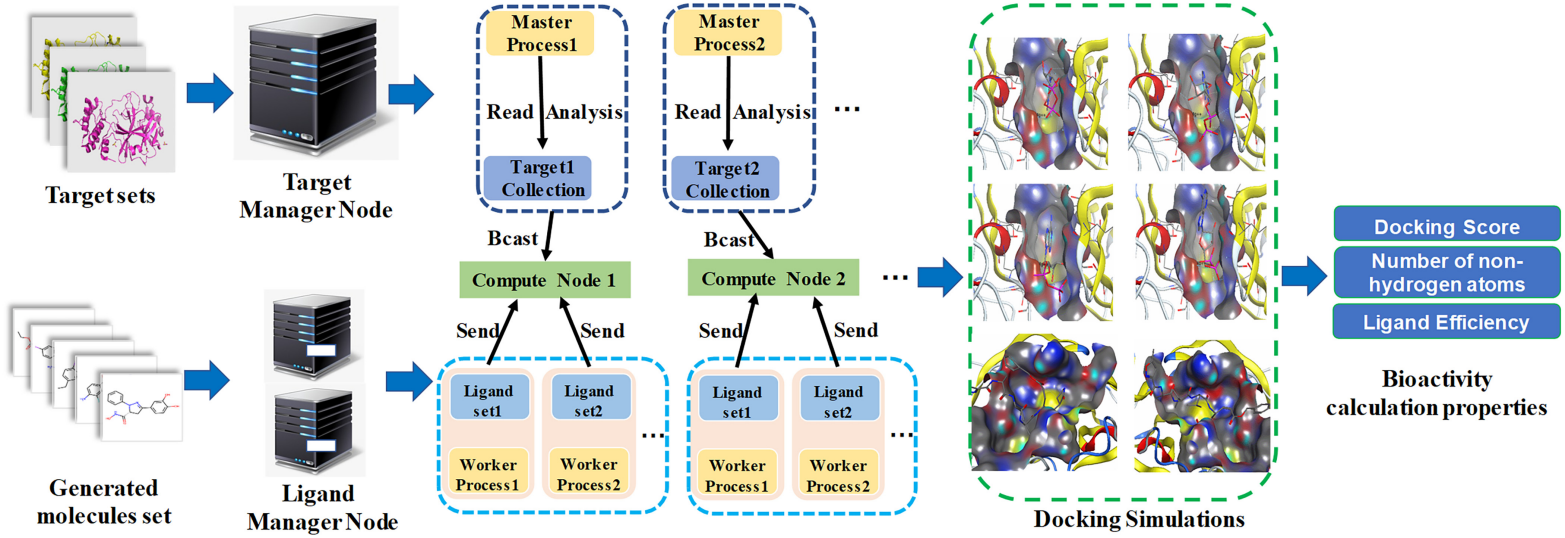
The specific task scheduling and allocation process.

Specifically, we first preprocessed ligand data and protein target files to create multiple ligand sets and specific target collections. a “target management node” was established to read all target files and docking pocket information in batches, which was then broadcasted to each computing node. Simultaneously, multiple “ligand management nodes” were assigned to read small molecule files and distributed them to computing nodes. Subsequently, molecular docking was performed between target files and small molecule files in the ligand sets, resulting in docking outcomes upon completion. Figure 3 shows the specific task scheduling and allocation process.

In the molecular optimization, the bioactivity prediction was realized by constructing classifiers. Initially, the ic50 value was used as the benchmark, with active molecules as positive samples and inactive molecules as negative samples. The perceptron generated the activity information by virtually docking between the data of positive and negative samples and disease-related targets. From the docking results, we extracted the docking score, number of nonhydrogen atoms, and ratio of the first two. These features were then input into the perceptron to predict activity. In addition, a large-scale molecular docking was employed to quickly and accurately calculate the affinity score between small molecules and specific targets, and to predict the real activity of generated molecules, so as to further optimize the biological activity.

In brief, the data obtained through molecular characterization was fed as the input to the model. The actor then adapted the generation strategy using transformer-DNN, with particular emphasis on molecular optimization being incorporated into the reward mechanism. Finally, critic calculated the value of the generation strategy adopted by the actor after receiving the reward. If yes, the actor’s action would be strengthened. If no, be blocked. Then, the current state was replaced by the new state. The actor was rewarded for each valid molecule generated. The reward was higher if it managed to produce molecules that met the expectations of multi-objective properties. The valid molecules generated were taken as the input for the next generation task. Through continuous iteration, the model accuracy was optimized to the maximum, which was marked by the stable proportion of high-quality molecules generated.

## 3 Experiments

### 3.1 Dataset and preprocessing

We utilized two common datasets to evaluate model performance. The first one was the MOSES dataset (Polykovskiy *et al.* 2020), composed of 1.9 million lead compounds filtering from the ZINC database, which had favorable drug-like properties. The second one was the GuacaMol dataset (Brown *et al.* 2019), which was a subset of ChEMBL 24, containing 1.6 million molecules with diverse distributions in terms of MW, LogP, TPSA and the number of rotatable bonds. We randomly selected 100 000 molecules for model training, and in the testing phase, we evaluated the model using 10 000 generated molecules.

Moreover, we constructed a dataset for case studies focusing on specific Mpro targets. The dataset was divided into two parts. One part was the initial compound group that contained 175 lead compounds, selected from 969 lead compounds by virtual screening. The other part was a virtual fragment library with 9823 Mpro inhibitory activity from the ChEMBL. And, in the reward mechanism of reinforcement learning, the biological activity prediction was realized by constructing perceptron classifiers. The perceptron model used the docking software Ledock to dock between the existing relevant PDB files of 380 different 3D conformations of Mpro targets and molecules generated in each epoch. In the pretraining process of the perceptron classifier, we used the dekois 2.0 benchmark dataset library provided by Tubingen University, which contained the SARS coronavirus 3CL protease dataset (Ibrahim *et al.* 2015).

In molecular optimization, we optimized several multi-objective properties, including LogP, PSA, MW, and toxicity. The target ranges for these properties were shown in Table 1. To obtain the reference range, we calculated properties values of 2086 patent drug published on the DrugBank (Wishart *et al.* 2018) using the ADMETLab2.0 (Xiong *et al.* 2021). We then made probability statistics as shown in Supplementary Figs S1–S4. According to the Lipinski rule and the empirical parameters guided by the statistical results of the combined drugs, the compounds falling within these reference ranges were likely to have better pharmacokinetic properties and higher bioavailability during metabolic processes in the organism.

**Table 1.** Targeted molecular properties and their desired value ranges.

Property	Minimal	Maximal
MW	200	500
LogP	1.5	5.5
TPSA	40	120
Toxicophores	0	2

### 3.2 Evaluation metrics

We used a set of metrics from the MOSES and GuacaMol benchmarks to assess the quality and diversity of the generated chemical compounds.

**Validity**: The proportion of molecules that satisfy chemical validity criteria. Higher validity means that the model has a better grasp of fundamental chemical rules.

**Novelty**: The ratio of generated valid molecules that do not exist in the training dataset, using to assess the model’s ability to generate novel molecules.

**Uniqueness**: The distinct components within the molecules generated effectively, aiding in assessing the model’s grasp of the molecular distribution in the data. Higher uniqueness means that the model can generate more diverse molecules.

**Internal diversity**: The similarity among molecules in the generated molecular set. As shown in Equation (8), *T* represents the Tanimoto similarity between molecules $\;m_{1}$ and $\;m_{2}$ in the generated set *G*. A higher value of this metric indicates higher diversity within the generated set.


(8) $${\text{IntDiv}}_{p}(G)=1-\sqrt[{p}]{\frac{1}{|G{|}^{2}}\sum_{m_{1},\;m_{2}\in G}T{\left(\;m_{1},\;m_{2}\right)}^{p}}$$

**Scaffold similarity**: Cosine similarity of Bemis–Murcko scaffold frequencies between two sets of compounds. Expressing $c_{s}(A)$ as the frequency of scaffold s appearing in molecules from set *A*, and defining *S* as the set of fragments found in either *G* or *R*, the metric is defined as shown in Equation (9):


(9) $$\text{Scaff}(G,R)=\frac{\sum_{s\in S}\left[c_{s}(G)\cdot c_{s}(R)\right]}{\sqrt{\sum_{s\in S}c_{s}^{2}(G)}\sqrt{\sum_{s\in S}c_{s}^{2}(R)}}$$

**Fréchet ChemNet distance (FCD)**: A distance measure that quantifies the chemical and biological similarity between two sets of compounds. As shown in Equation (10), $\mu_{G}$, $\mu_{R}$ are mean vectors and $\Sigma_{G}$, $\Sigma_{R}$ are full covariance matrices of activations for molecules from sets *G* and *R* respectively.


(10) $$\begin{array}{c} \text{FCD}(G,R)={\left\|\mu_{G}-\mu_{R}\right\|}^{2}+\text{Tr}(\Sigma_{G}+\Sigma_{R}\\ -2{\left(\Sigma_{G}\Sigma_{R}\right)}^{1/2}) \end{array}$$

### 3.3 Method comparison

To assess our method, we generated 10 000 molecules by sampling and benchmarked the model using several different molecular generation methods based on the MOSES and GuacaMol datasets except activity, which including non-neural baselines [n-gram generative model and Hidden Markov Model (HMM)], JTN-VAE (Jin *et al.* 2018), CharRNN (Segler *et al.* 2018), AAE (Polykovskiy *et al.* 2018), LatentGAN (Prykhodko *et al.* 2019), Organ (Guimaraes *et al.* 2017), VAE (Gómez-Bombarelli *et al.* 2018), and MoFlow (Zang and Wang 2020).

We conducted molecular generation on the NOSES dataset and the GuacaMol dataset, sampling 10 000 molecules and counting various metrics for the generated molecules. Table 2 shows the performance of the baseline and our model on the MOSES dataset, indicating that our model achieves optimal novelty and diversity. However, due to the uneven probability distribution of fragment selection and replacement, the validity of our model was slightly lower. In contrast, JTVAE achieved 100% validity due to its tree decomposition and predefined subgraphs, avoiding learning SMILES syntax, but its other three indicators were not prominent. MoFlow also attained 100% validity (thanks to validity checking components) and scaffold similarity, but the internal diversity was lower, which meant that the model cannot effectively modify the scaffold and was less suitable as a scaffold transformation.

**Table 2.** Performances metrics of different models on the MOSES dataset with sampling 10 000 molecules generated.

Models	Validity	Novelty	Int-div	Scaf-sim
HMM	0.076	0.999	0.810	0.206
Ngram	0.238	0.969	0.864	0.530
AAE	0.937	0.793	0.856	0.902
CharRNN	0.975	0.842	0.856	0.924
JTN-VAE	**1.0**	0.915	0.845	0.892
LatentGAN	0.897	0.949	0.857	0.886
MoFlow	**1.0**	0.996	0.617	**1.0**
Ours	0.950	**0.999**	**0.872**	0.930

The bold values signify the highest value for that metric.

Table 3 displays the performance of the baseline and our model on the GuacaMol dataset. SMILES LSTM, AAE, and VAE exhibited strong validity, uniqueness, and novelty scores (>80%). Conversely, Organ underperformed significantly, demonstrating lower validity (37.9%), novelty (68.7%), and an FCD value of 0. This indicated that Organ struggled to effectively learn the data distribution of the training set and failed to adequately consider the molecular scaffold structure. AAE overcaptured features from the training data, resulting in similar molecules in the embedding space being closer, leading to lower FCD values. In contrast, our model had achieved the best performance in all aspects, especially achieving 100% uniqueness and novelty This conclusively demonstrated that our model outperforms others in overall performance.

**Table 3.** Performances metrics of different models on the GuacaMol dataset with sampling 10 000 molecules generated.

Models	Validity	Uniqueness	Novelty	FCD
SMILES LSTM	0.959	**1.0**	0.912	0.913
AAE	0.822	**1.0**	0.998	0.529
Organ	0.379	0.841	0.687	0
VAE	0.870	0.999	0.974	0.863
Ours	**0.963**	**1.0**	**1.0**	**0.937**

The bold values signify the highest value for that metric.

### 3.4 Target-specific molecules generation

For the specific Mpro target, we introduced an activity optimization strategy. Utilizing the dataset we had created, we successfully generated high-quality molecular compounds, offering promising candidates for anti-PEDV to the Shandong University School of Pharmacy. Furthermore, we also conducted a comprehensive evaluation, covering various critical indicators, including analyzing the property distribution, similarity, synthetic accessibility (SA), and drug-likeness, as well as the binding model of the generated molecule and its corresponding crystal structure, to ensure that these molecules had good potential and feasibility in anti-PEDV drug research.

**Figure 4.**
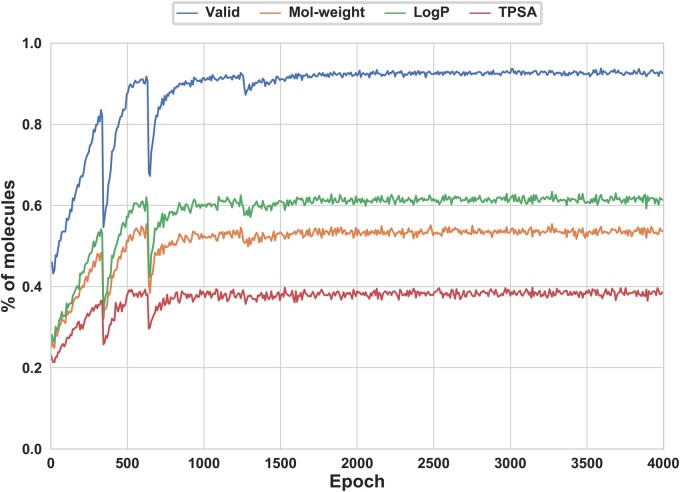
The percentage of valid molecules and the percentage of molecules that fulfill the target ranges specified in Table 1.

**Molecular properties**: We focused on optimizing four key properties, namely effectiveness, logP, PSA and MW. The percentage of generated molecules that met the target ranges for these properties was shown in Fig. 4. Overall, with the increase of epoch, the proportion of molecules that satisfied the target ranges for each property increased and tended to be stable. In the end, among the molecules we had successfully generated, nearly 98% were effective compounds, with over 40% of them meeting all desired properties.

In addition, we counted the properties distribution of MW, logP and PSA. Compared with the distribution in the initial set, the distribution of target properties generated in the last 10 epochs was shown in Supplementary Figs. S5–S7. It could be found that property distribution of the generated molecular sets (red) closely matched that of the initial lead molecule set and were within the target properties range specified in Table 1.

**Figure 5.**
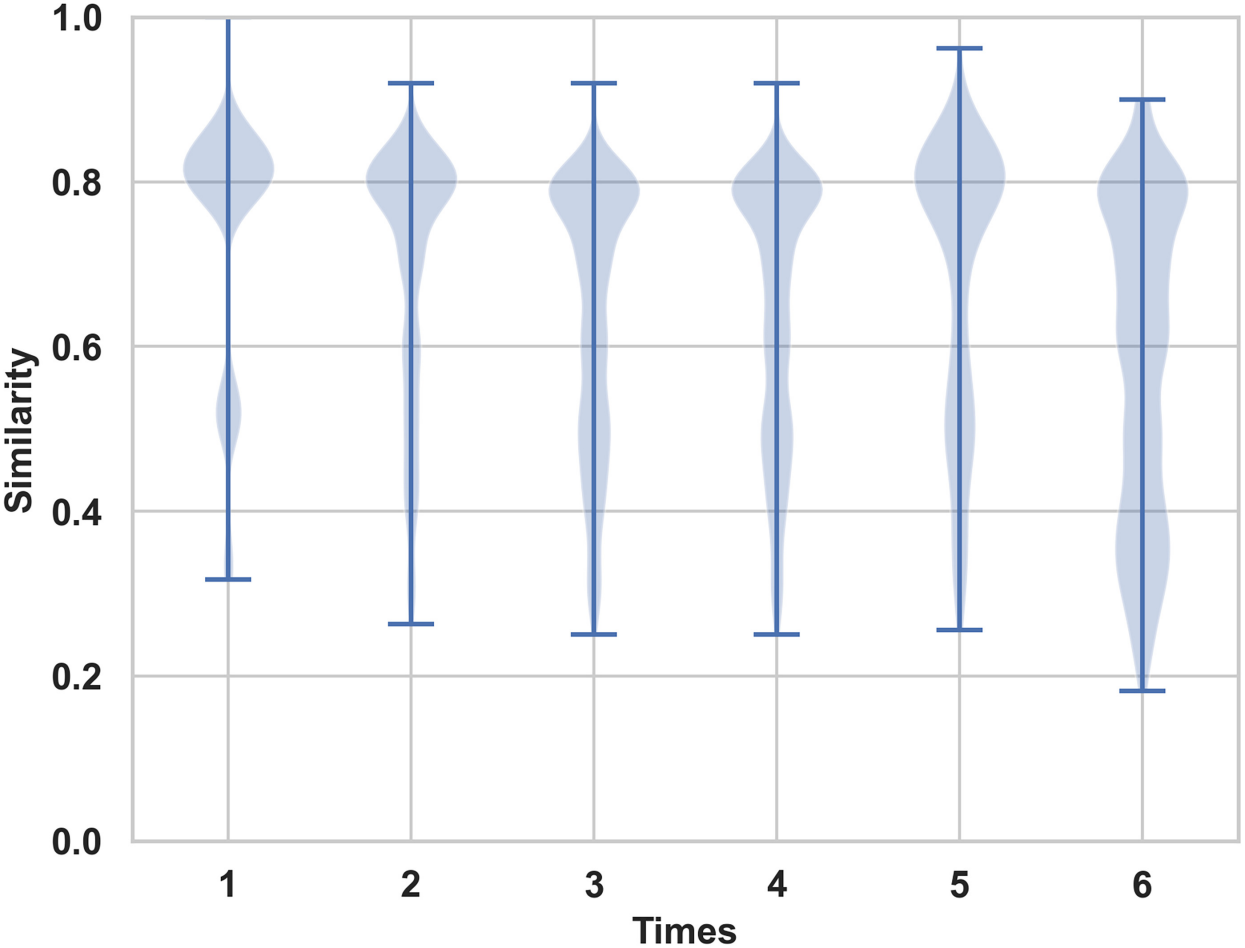
The distribution of similarity between the generated and the initial molecules.

**Molecular similarity**: The similarity between newly molecules and the initial molecules remained stable at 0.8 under different Time parameters. As shown in Fig. 5, with the increase of the parameter, that was, the increase of replacement fragments, the similarity values of the generated new molecules and the original molecules scattered in different intervals. This indicated the increasing number of replaced molecular fragments led to the increase in the novelty of the newly generated molecules. This could be attributed to the fact that the initial molecular structures underwent multiple modifications, resulting in a greater diversity and novelty in the generated molecular structures.

**Figure 6.**
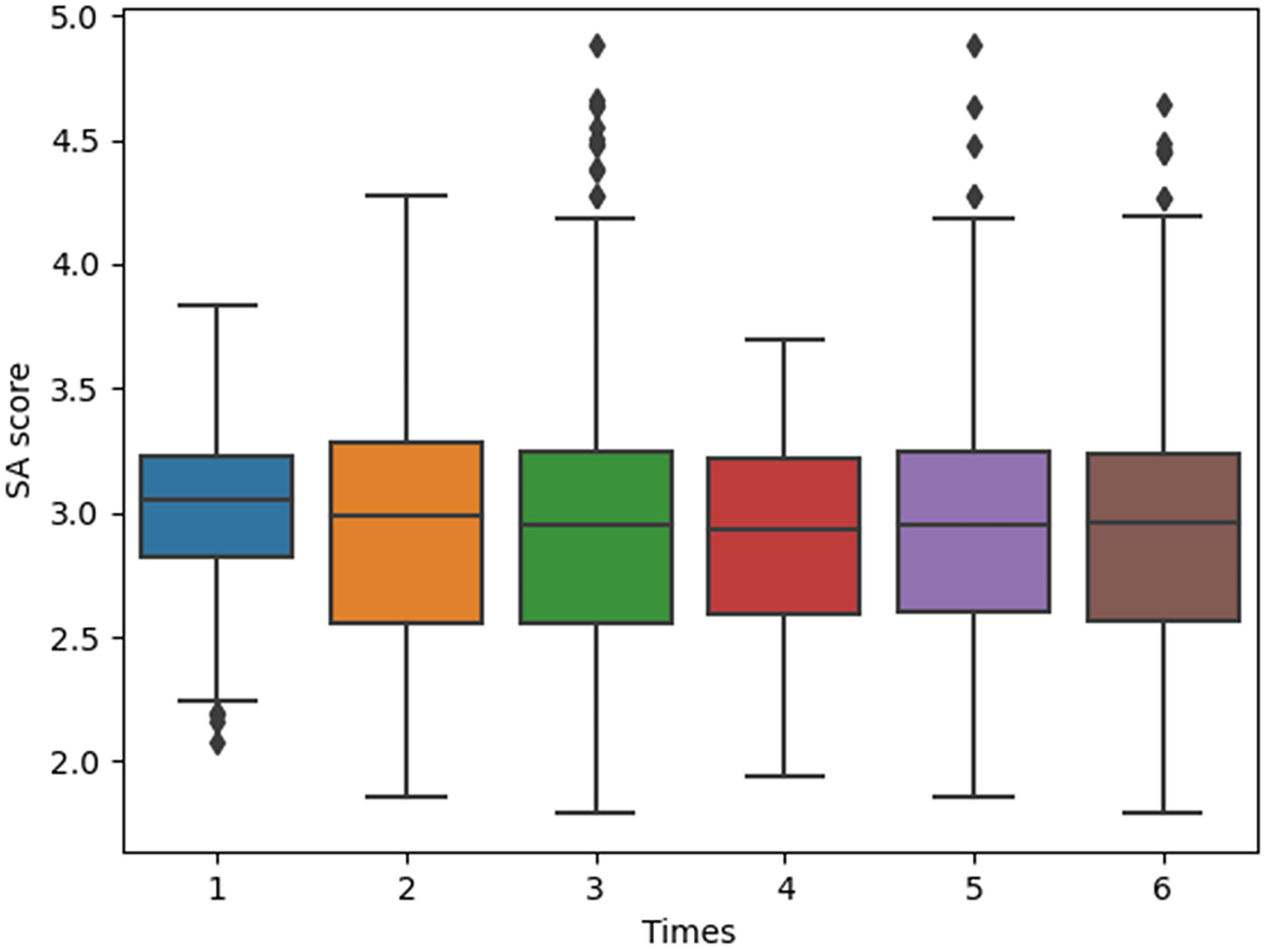
The distribution of SA score of generated molecules.

**Synthetic accessibility**: SA score was used to assess the difficulty of compound synthesis. We analyzed the SA score distribution of the generated molecules in Fig. 6. Except for parameter 3, which had a relatively high number of outliers, the molecular SA property distributions generated by the other parameters were mostly within the 1.5 interquartile range (IQR). In addition, the median of the SA properties was consistently close to 3, with a range of 1 to 10, where values closer to 1 indicated molecules that were easier to synthesize.

**Figure 7.**
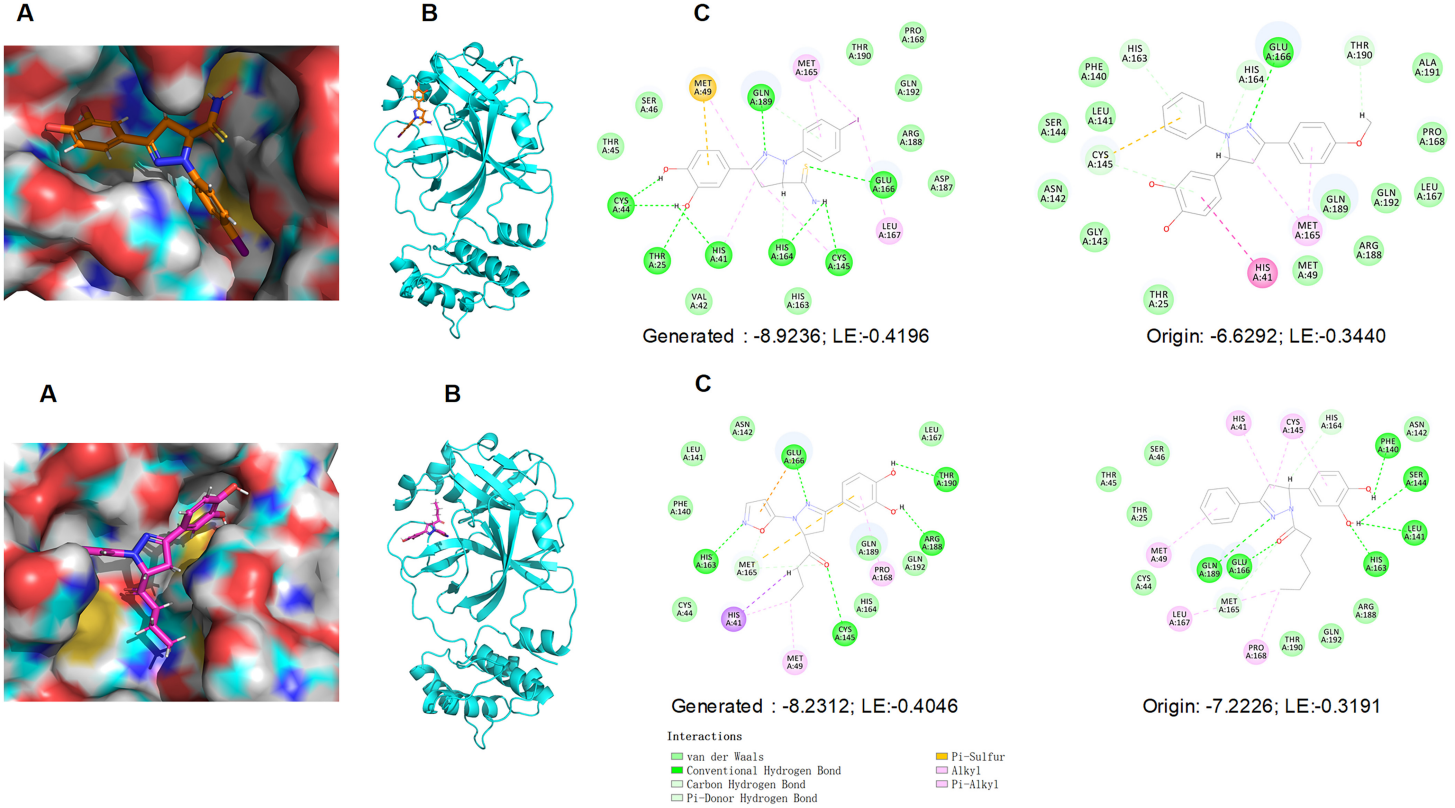
Binding model of the generated molecule and its crystal structure. A, B, and C correspond to 6*L*70. C shows the protein–ligand interactions of the known compounds and the generated molecules.

**Binding model**: We selected molecules with highest binding energy and ligand efficiency from the generated molecules and analyzed the binding model of the generated molecule and its crystal structure with SARS-CoV-2 Mpro of COVID-19. Then, we compared the binding energy and ligand efficiency of the generated molecules with their corresponding initial molecules. As shown in Fig. 7, we observed that the optimal docking score obtained for the generated molecules was superior to that of the known compounds.

**Figure 8.**
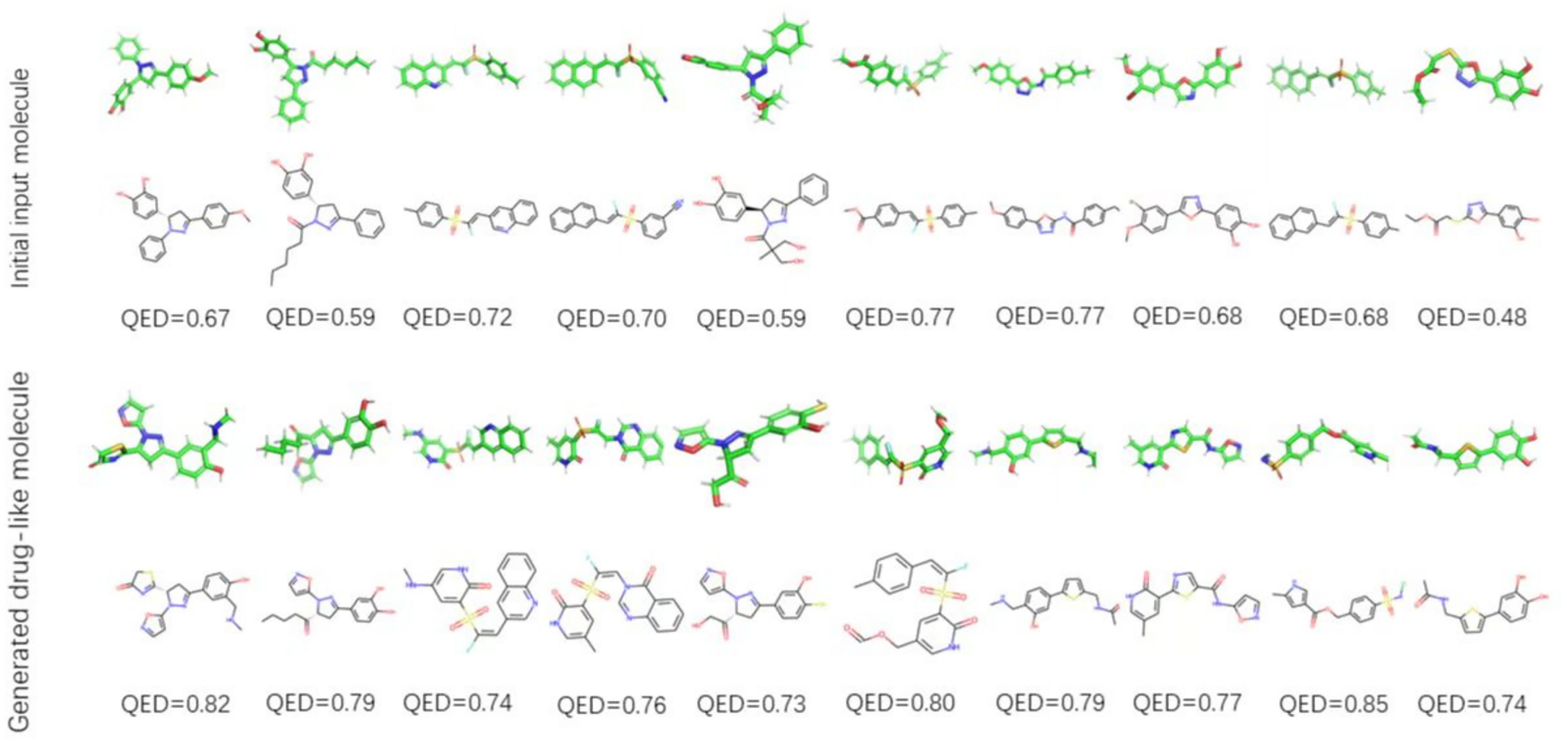
The change of QED score between the generated and the initial molecules.

**Drug-likeness**: Drug-likeness (QED) scores were a quantitative estimate to evaluate the similarity between the generated compound molecules and known drugs. We randomly selected 10 molecules with better binding energy and ligand efficiency from the generated molecules and analyzed the change of QED score between the generated and the initial molecules. As shown in Fig. 8, the QED scores of the generated molecules was greater than that of the initial molecules, and where the QED score was >0.7.

### 3.5 Ablation studies

To validate the impact of properties optimization on generating high-quality molecules, we conducted a series of ablation experiments and analyzed validity, novelty, internal diversity as well as scaffold similarity of the generated molecules. As shown in Table 4, the molecules generated in the model with properties optimization exhibited a significantly improved validity, rising from 0.820 to 0.950. Furthermore, more satisfactory results were achieved in terms of novelty (0.999 versus 0.869) and scaffold similarity (0.930 versus 0.785). In contrast, the impact of whether properties optimization was employed within the model on internal diversity was not as evident. This might be attributed to the fact that the objectives of properties optimization tend to ensure that the generated molecules meet specific attributes or rules, resulting in relatively consistent internal structures and a reduction in internal diversity.

**Table 4.** The impact of properties optimization on the model.

Properties	Validity	Novelty	Int-div	Scaf-sim
With	0.950	0.999	0.872	0.930
Without	0.820	0.869	0.870	0.785

## 4 Conclusion

Improving the efficiency and quality of molecule design is crucial in the process of the development of innovative drugs. The key is to balance the bioactivity and multi-physicochemical properties based on molecular scaffolds, which is difficult to achieve through opportunistic compound screening and artificial structure optimization. In this study, we proposed a novel molecular generation strategy and optimization based on A2C reinforcement learning. In molecular generation strategy, we adopted transformer-DNN to retain the scaffolds advantages, while accounting for the generated molecules’ similarity and internal diversity by dynamic parameter adjustment, further improving the overall quality of molecule generation. In molecular optimization, we introduced heterogeneous parallel supercomputing for large-scale molecular docking based on MPI communication technology to rapidly obtain bioactive information, thereby enhancing the efficiency of drug design. On this basis, future research can be extended to the characterization based on graph with 3D geometric information. In molecular generation strategies, it is possible to consider the introduction of diffusion models or their combination with larger models to optimize drug molecule design. For molecular optimization, the ADMET properties of molecules can also be considered in future research to facilitate the simplification of downstream molecular synthesis and drug measurement. Moreover, our method may provide important insights into the refinement of molecule generation and optimization for self-learning automatic molecular design.

## Supplementary Material

btad693_Supplementary_Data

## References

[btad693-B1] Bjerrum EJ , Sattarov B. Improving chemical autoencoder latent space and molecular de novo generation diversity with heteroencoders. Biomolecules 2018;8:131. 30380783 10.3390/biom8040131 PMC6316879

[btad693-B2] Blaschke T , Engkvist O, Bajorath J et al Memory-assisted reinforcement learning for diverse molecular de novo design. J Cheminform 2020;12:68. 33292554 10.1186/s13321-020-00473-0 PMC7654024

[btad693-B3] Brown N , Fiscato M, Segler MHS et al Guacamol: benchmarking models for de novo molecular design. J Chem Inf Model 2019;59:1096–108. 30887799 10.1021/acs.jcim.8b00839

[btad693-B4] Cherti M , Kégl B, Kazakçı A. De novo drug design with deep generative models: an empirical study. In: *International Conference on Learning Representations*, Toulon, France, April 24–26, 2017. 2017.

[btad693-B5] Dai H , Tian Y, Dai B et al Syntax-directed variational autoencoder for structured data. arXiv, arXiv:1802.08786, 2018, preprint: not peer reviewed. 10.48550/arXiv.1802.08786

[btad693-B6] De Cao N , Kipf T. Molgan: an implicit generative model for small molecular graphs. In: *ICML 2018 Workshop*, Stockholm, Sweden. PMLR 80, 2018.

[btad693-B7] Dong Y , Li D, Zhang C et al Inverse design of two-dimensional graphene/H-BN hybrids by a regressional and conditional GAN. Carbon 2020;169:9–16.

[btad693-B8] Gómez-Bombarelli R , Wei JN, Duvenaud D et al Automatic chemical design using a data-driven continuous representation of molecules. ACS Cent Sci 2018;4:268–76. 29532027 10.1021/acscentsci.7b00572 PMC5833007

[btad693-B9] Guimaraes G , Sanchez-Lengeling B, Outeiral C et al Objective-reinforced generative adversarial networks (organ) for sequence generation models. arXiv, arXiv:1705.10843, 2017, preprint: not peer reviewed. 10.48550/arXiv.1705.10843

[btad693-B10] Ibrahim T , Bauer MR, Dörr A et al pROC-chemotype plots enhance the interpretability of benchmarking results in structure-based virtual screening. J Chem Inf Model 2015;55:2297–307. 26434782 10.1021/acs.jcim.5b00475

[btad693-B11] Jin W , Barzilay R, Jaakkola T et al Junction tree variational autoencoder for molecular graph generation. In: *Proceedings of the 35th International Conference on Machine Learning*, Stockholm, Sweden, Vol. 80. PMLR, 2018, 2323–32.

[btad693-B12] Kingma DP , Welling M. An introduction to variational autoencoders. FNT Mach Learn 2019;12:307–92.

[btad693-B13] Li J , Topaloglu RO, Ghosh S et al Quantum generative models for small molecule drug discovery. IEEE Trans Quantum Eng 2021;2:1–8.

[btad693-B14] Li Y , Zhang L, Liu Z et al Multi-objective de novo drug design with conditional graph generative model. J Cheminform 2018;10:33. 30043127 10.1186/s13321-018-0287-6 PMC6057868

[btad693-B15] Luo S , Guan J, Ms J et al A 3D generative model for structure-based drug design. Adv Neural Inform Process Syst 2021;34:6229–39.

[btad693-B16] Mercado R , Rastemo T, Lindelöf E et al Graph networks for molecular design. Mach Learn Sci Technol 2021;2:025023.

[btad693-B17] Merk D , Friedrich L, Grisoni F et al De novo design of bioactive small molecules by artificial intelligence. Mol Inform 2018;37:1700153. 29319225 10.1002/minf.201700153 PMC5838524

[btad693-B18] Moret M , Friedrich L, Grisoni F et al Generative molecular design in low data regimes. Nat Mach Intell 2020;2:171–80.

[btad693-B19] Olivecrona M , Blaschke T, Engkvist O et al Molecular de-novo design through deep reinforcement learning. J Cheminform 2017;9:48. 29086083 10.1186/s13321-017-0235-x PMC5583141

[btad693-B20] Polykovskiy D , Zhebrak A, Vetrov D et al Entangled conditional adversarial autoencoder for de novo drug discovery. Mol Pharm 2018;15:4398–405. 30180591 10.1021/acs.molpharmaceut.8b00839

[btad693-B21] Polykovskiy D , Zhebrak A, Sanchez-Lengeling B et al Molecular sets (moses): a benchmarking platform for molecular generation models. Front Pharmacol 2020;11:565644. 33390943 10.3389/fphar.2020.565644 PMC7775580

[btad693-B22] Popova M , Isayev O, Tropsha A et al Deep reinforcement learning for de novo drug design. Sci Adv 2018;4:eaap7885. 30050984 10.1126/sciadv.aap7885 PMC6059760

[btad693-B23] Prykhodko O , Johansson VS, Kotsias P-C et al A de novo molecular generation method using latent vector based generative adversarial network. J Cheminf 2019;11:1–13. 10.1186/s13321-019-0397-9 PMC6892210 33430938

[btad693-B24] Ragoza F , Masuda t, Koes DR et al Learning a continuous representation of 3D molecular structures with deep generative models. In: *Machine Learning for Structural Biology Workshop, NeurIPS 2020*, Vancouver, Canada. 2020.

[btad693-B25] Scarselli F , Gori M, Tsoi AC et al The graph neural network model. IEEE Trans Neural Netw 2008;20:61–80. 19068426 10.1109/TNN.2008.2005605

[btad693-B26] Schütt KT , Arbabzadah F, Chmiela S et al Quantum-chemical insights from deep tensor neural networks. Nat Commun 2017;8:13890. 28067221 10.1038/ncomms13890 PMC5228054

[btad693-B27] Segler MHS , Kogej T, Tyrchan C et al Generating focused molecule libraries for drug discovery with recurrent neural networks. ACS Cent Sci 2018;4:120–31. 29392184 10.1021/acscentsci.7b00512 PMC5785775

[btad693-B28] Simonovsky M , Komodakis N. Graphvae: towards generation of small graphs using variational autoencoders. In: *Artificial Neural Networks and Machine Learning–ICANN 2018: 27th International Conference on Artificial Neural Networks, Rhodes, Greece, October 4–7, 2018, Proceedings, Part I 27*. Springer, 2018, 412–22.

[btad693-B29] Valverde JR. Molecular modelling: principles and applications. Brief Bioinf 2001;2:199–200.

[btad693-B30] Wang Q et al Deep reinforcement learning and docking simulations for autonomous molecule generation in de novo drug design. In: ACM Multimedia Asia, Gold Coast, Australia. New York, NY, USA: ACM, 2021, 1–6.

[btad693-B31] Wishart DS , Feunang YD, Guo AC et al Drugbank 5.0: a major update to the drugbank database for 2018. Nucleic Acids Res 2018;46:D1074–82. 29126136 10.1093/nar/gkx1037 PMC5753335

[btad693-B32] Xiong G , Wu Z, Yi J et al Admetlab 2.0: an integrated online platform for accurate and comprehensive predictions of admet properties. Nucleic Acids Res 2021;49:W5–14. 33893803 10.1093/nar/gkab255 PMC8262709

[btad693-B33] Yu L , Zhang W, Wang J et al Seqgan: sequence generative adversarial nets with policy gradient. In: *Proceedings of the Thirty-First AAAI Conference on Artificial Intelligence*, San Francisco, California. AAAI Press, Vol. 31, 2017.

[btad693-B34] Zang C , Wang F. MoFlow: an invertible flow model for generating molecular graphs. In: *Proceedings of the 26th ACM SIGKDD International Conference on Knowledge Discovery & Data Mining*, CA, USA. New York, NY, USA: ACM, 2020, 1–10.

