# Supplementary material for: Molecular generation strategy and optimization based on A2C reinforcement learning in de novo drug design
Source: Bioinformatics. 2023 Nov 16;39(11):btad693. doi: 10.1093/bioinformatics/btad693 (PMC10689670; doi:10.1093/bioinformatics/btad693)
Supplement: btad693_Supplementary_Data [file btad693_supplementary_data.docx]

**Molecular generation strategy and optimization based on** **A2C reinforcement learning in** **de novo** **drug design**

Qian Wang^1†^, Zhiqiang Wei^1†^, Xiaotong Hu^1^, Zhuoya Wang^2^, Yujie Dong^3^, Hao Liu^1*^

^1^College of Computer Science and Technology, Ocean University of China, Qingdao, 266003, Shandong, China. ^2^Center for High Performance Computing and System Simulation, National Laboratory for Marine Science and Technology, Qingdao, 266237, Shandong, China. ^3^Marine Big Data Center of Institute for Advanced Ocean Study, Ocean University of China, Qingdao, 266003, Shandong, China.

†Equal contribution

*Corresponding author: Hao Liu. E-mail: [liu.hao@ouc.edu.cn](mailto:liu.hao@ouc.edu.cn)

To obtain the reference range, we calculated properties values of 2086 patent drug published on the DrugBank using the ADMETLab2.0. We then made probability statistics as shown in Fig.S1-S4.


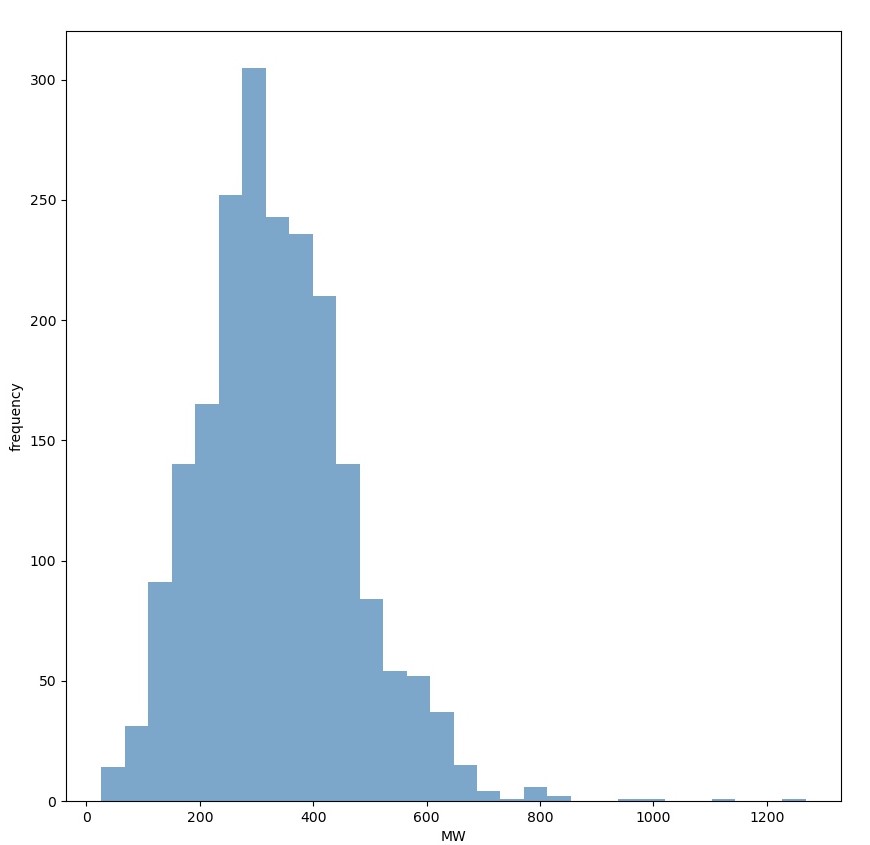


Fig.S1. The probability statistics of molecular weight property ranges


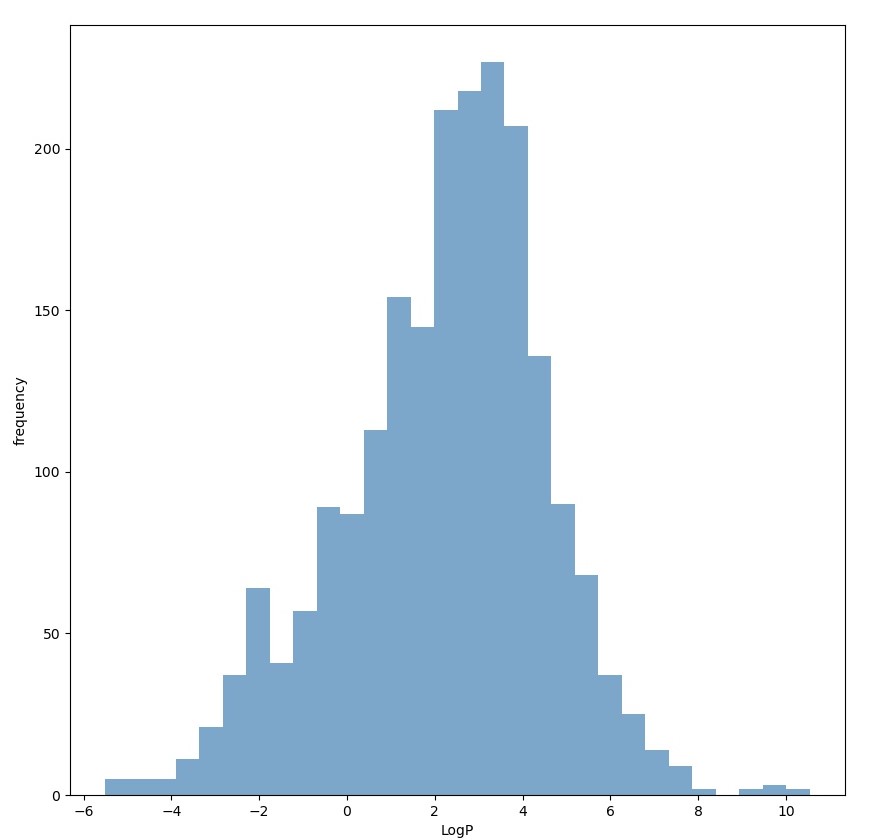


Fig.S2. The probability statistics of LogP property ranges


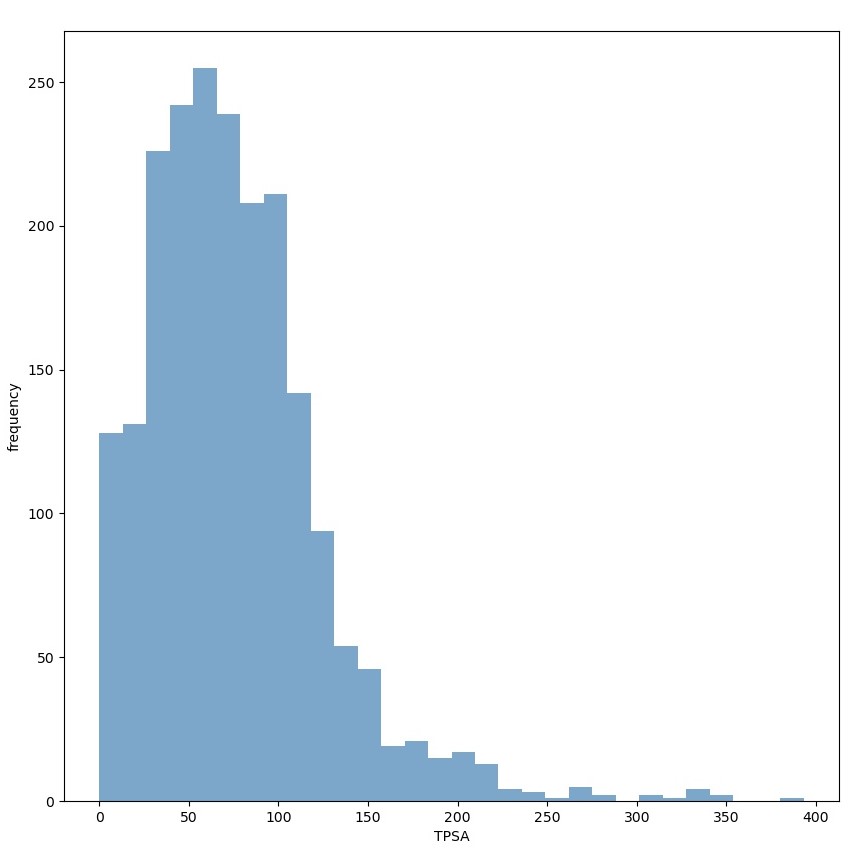


Fig.S3. The probability statistics of TPSA property ranges


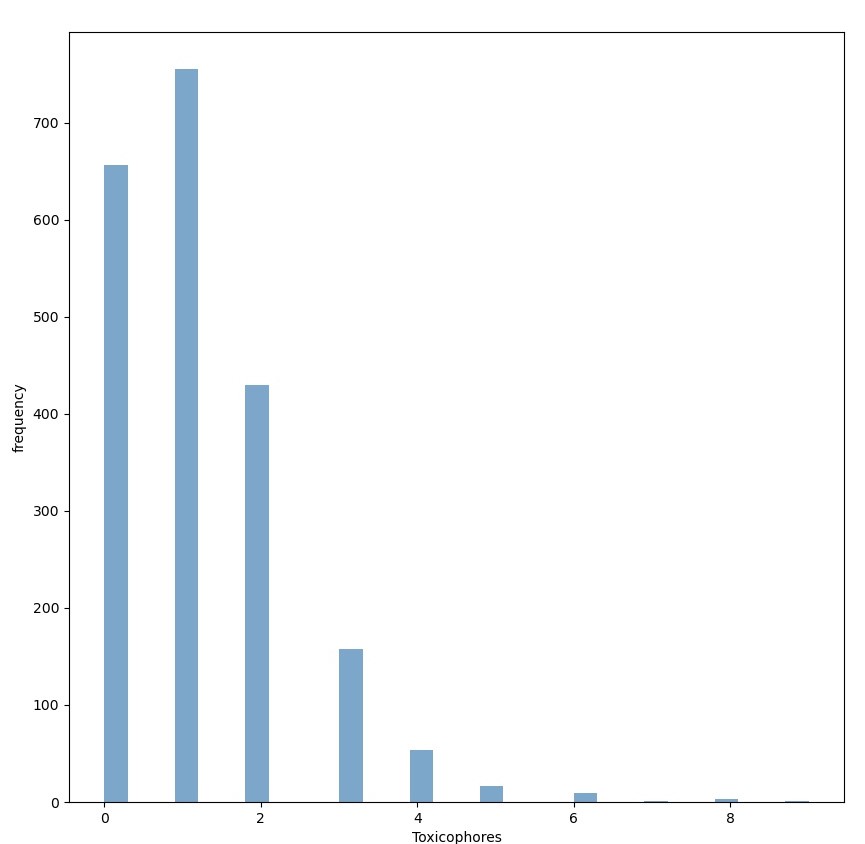


Fig.S4. The probability statistics of TPSA property ranges

We counted the properties distribution of molecular weight, lipophilicity and polar surface area. Compared with the distribution in initial lead set, the distribution of target properties generated in the last 10 epochs was shown in Supplementary Fig.S5-S7.


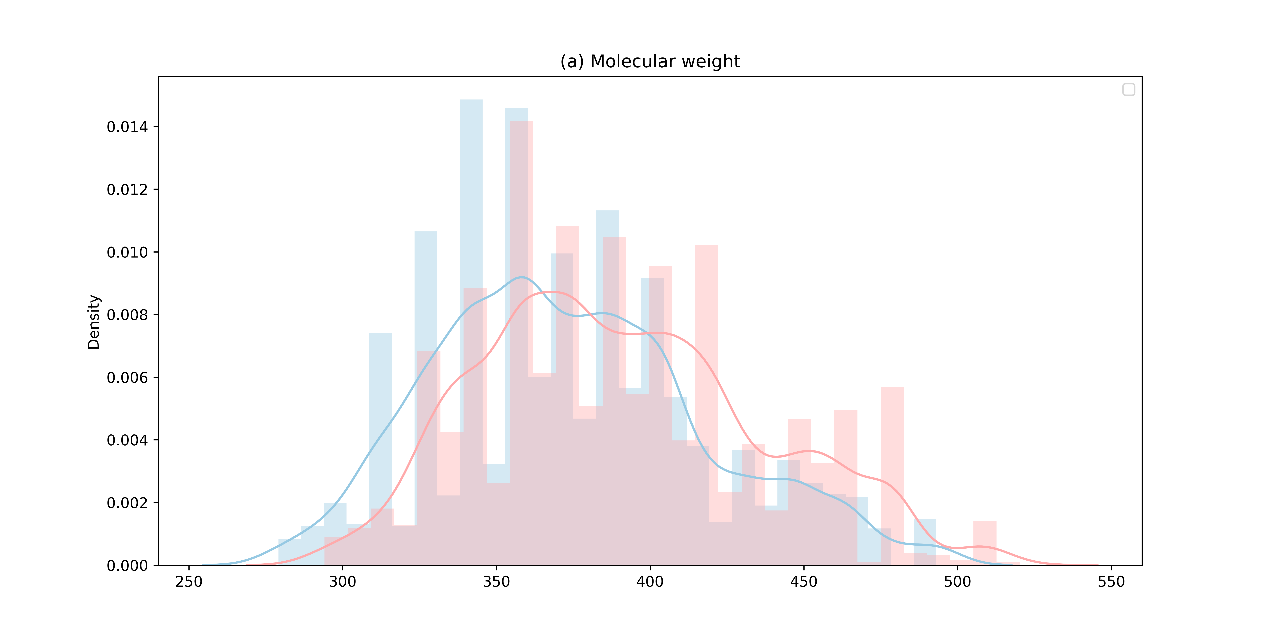


Fig. S5. The distribution of molecular weight among the lead molecules

(blue) and the generated molecules (red)


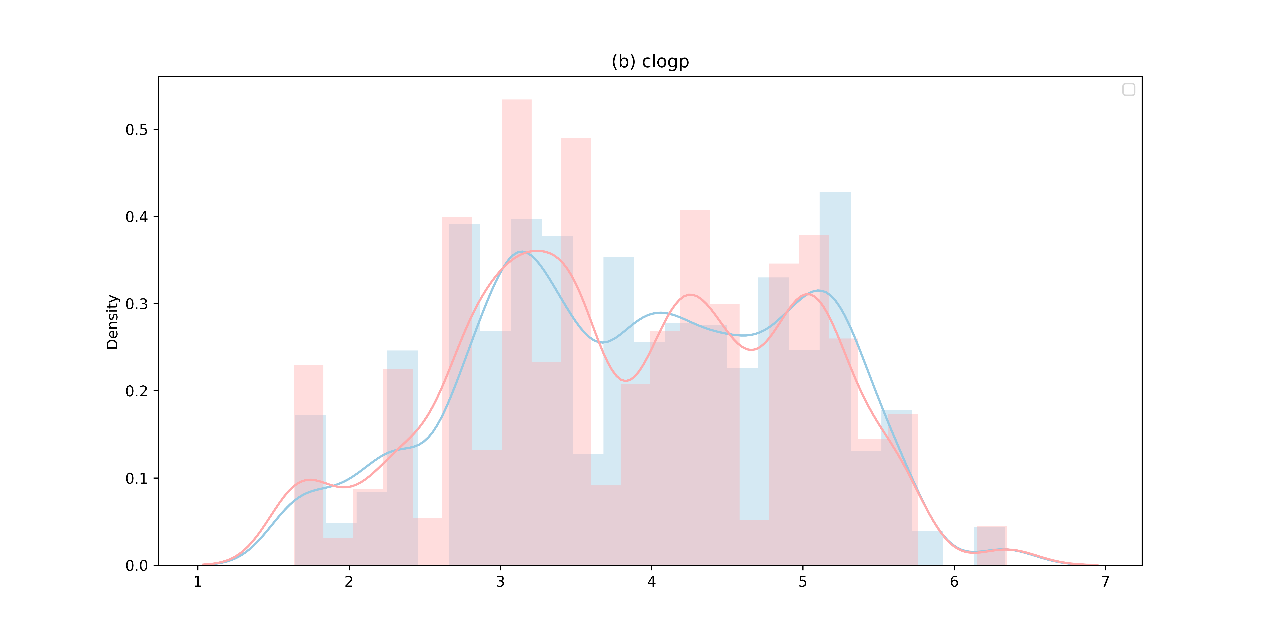


Fig. S6. The distribution of clogp among the lead molecules

(blue) and the generated molecules (red)


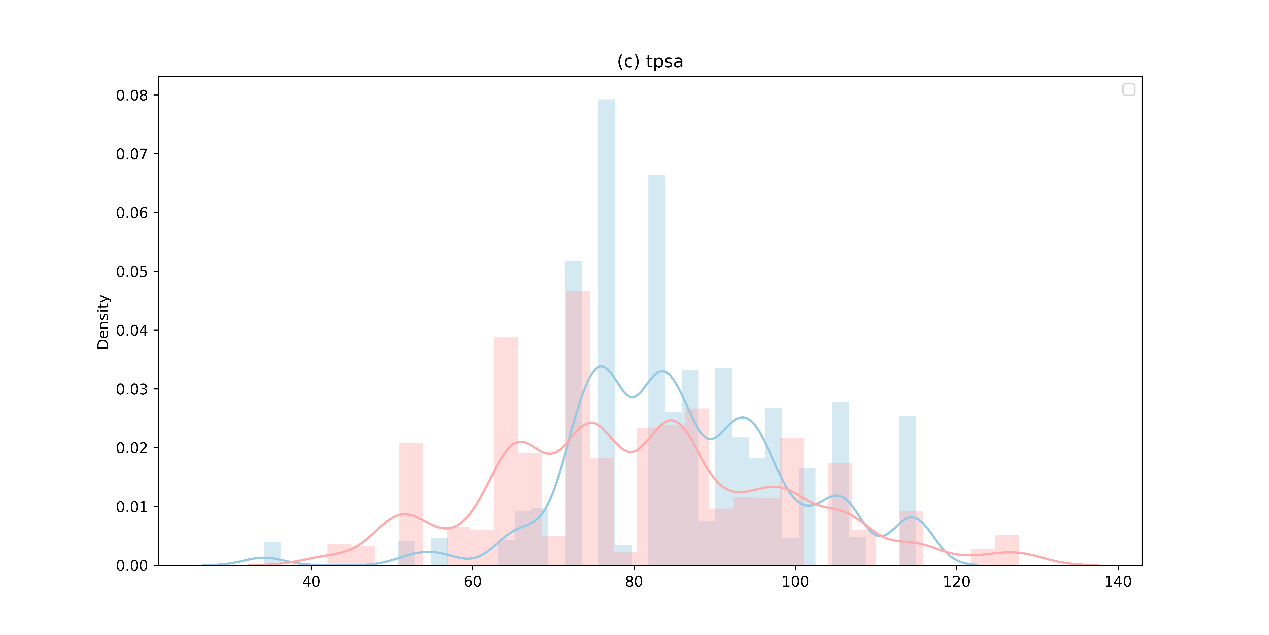


Fig. S7. The distribution of tpsa among the lead molecules

(blue) and the generated molecules (red)
